# Transnational practices of Kazakh repatriates: the role of family in the adaptation of ethnic Kazakh students from Mongolia and China

**DOI:** 10.3389/fsoc.2024.1452785

**Published:** 2024-09-13

**Authors:** Sholpan Zharkynbekova, Saule Tazhibayeva, Zukhra Shakhputova, Zhazira Agabekova, Ariya Azamatova, Zhanna Kuzar

**Affiliations:** ^1^Department of Theoretical and Applied Linguistics, L.N. Gumilyov Eurasian National University, Astana, Kazakhstan; ^2^Department of Theory and Practice of Translation, L.N. Gumilyov Eurasian National University, Astana, Kazakhstan; ^3^Department of Foreign Languages, L.N. Gumilyov Eurasian National University, Astana, Kazakhstan; ^4^Department of Kazakh Language and Turkic Studies, Nazarbayev University, Astana, Kazakhstan; ^5^Department of Modern Languages and Translation, Kazakhstan Institute of Management, Economics and Strategic Research (KIMEP) University, Almaty, Kazakhstan; ^6^Department of Foreign Languages, L.N. Gumilyov Eurasian National University, Astana, Kazakhstan

**Keywords:** transnational migration, transnational family, repatriate students from China and Mongolia, educational space, socio-cultural adaptation

## Abstract

Migration processes, which intensified in the late twentieth and early twenty-first centuries, occur within a context of constant challenges and changing realities, necessitating new research in this area. Nearly all migrants, adapting to new forms of everyday existence, might experience socio-psychological stress. This study focuses on the socio-cultural and linguistic adaptation of the young generation of ethnic Kazakhs, as well as their psychological state within the educational environment, grounded in the concept of transnationalism. The authors conducted a survey and interviews with repatriate students from China and Mongolia in their native language, Kazakh. The research methodology, survey methodology and applied tools are comparable in terms of both qualitative and quantitative analysis. The sample population consisted of 230 respondents from five regions, aged between 16 and 25 years old. Furthermore, 30 qualitative, focused interviews were conducted. The discussion questions are related to several important factors, including the impact of the presence of relatives in the country of origin, the establishment of ties with them, and the integration of individuals into the social spheres of their historical homeland during their stay in Kazakhstan, as well as the processes of adaptation and integration into the new society. The results demonstrate the existence of transnational practices among the informants, which permits the categorization of these individuals as transnational migrants, despite the fact that a considerable number of them have completed the migration process. The findings of the research also indicated that repatriate students from Mongolia showed greater ease of adaptation in Kazakhstan, largely due to their more active communication with relatives and stronger inclination toward integration into local society. Conversely, Kazakh ethnic students from China, despite using contemporary communication technologies, encountered more challenges, largely due to emotional difficulties associated with the lack of physical proximity to their families and a prevalent intention to return to their families after graduation.

## 1 Introduction

Migration is a significant phenomenon in the modern world, presenting a range of challenges for those who undertake it. Migrants face difficulties such as adapting to new customs, laws, and ways of life, while also overcoming cultural, linguistic, social, and economic barriers. The intensification of migration in our increasingly mobile and globalized world, particularly in recent decades, has given rise to numerous socio-cultural and socio-psychological issues.

The return of ethnic Kazakhs from various parts of the world to their historical homeland has increasingly attracted researchers' attention. This interest spans the social, psychological, and linguistic adjustments of these returnees, as well as the formation and preservation of various types of identity. The life practices of ethnic Kazakhs, viewed through the lens of transnationalism theory, are of particular research interest.

This study focuses on investigating the influence of family ties on young ethnic Kazakhs from Mongolia and China who have migrated to Kazakhstan for educational purposes. The relevance of this research topic is underscored by the concept of “transnational family” within the broader framework of transnationalism, which suggests a new methodological approach to the study of migrants' lives.

Understanding the transformation of family ties among ethnic Kazakh students from Mongolia and China and their impact on the process of adaptation in Kazakhstan, which is currently insufficiently studied, will make a significant contribution to migration studies. This study is based on field materials from a research project dedicated to the integration of ethnic Kazakhs from China and Mongolia into the Republic of Kazakhstan. Within this study, the authors address the following questions:

1. What are the dynamics of relationships within the families of migrant students and their parents residing in China and Mongolia?
2. How do these family relationships influence the adaptation and integration of ethnic Kazakh students from China and Mongolia?
3. What is the role of family in the emotional support of ethnic Kazakh students, and how does it influence their behavioral changes?

The historical foundation of this research is based on empirical data and legal acts regulating migration processes, which have enabled a detailed description of the socio-cultural, demographic, and economic factors influencing the adaptation process of repatriates from China and Mongolia over the past 30 years. The sociolinguistic approach involved conducting questionnaires and interviews with ethnic Kazakh students from Mongolia and China to accurately describe the dynamics of parent-child relationships.

The research methodology draws on the works of Glick Schiller et al. (1995), Baldassar (2008), Bryceson and Vuorela (2002), Macpherson and Macpherson (2009), Harris et al. (2020), Merla et al. (2021), Ngan and Chan (2022), and Zabrodskaja et al. (2023), which propose a systemic conceptualization of transnational practices based on a multidisciplinary approach. Despite the extensive scientific literature on both theoretical and methodological approaches to the study of ethnic repatriation and the socio-cultural and linguistic adaptation of Kazakh compatriots, there has been no research considering the ethnic Kazakh family as a transnational unit.

The transnational approach employed in this study allowed the authors to demonstrate the role of the family in the moral and psychological adaptation of students who relocated to Kazakhstan for their studies, as well as to identify the social behavior patterns of young repatriates in an unfamiliar cultural environment. The comparison and contrast of various interview texts and narratives in the form of biographical essays permitted the observation of the transformation of family and kinship ties operating in a transnational format.

## 2 Literature review

### 2.1 Transnational approach in contemporary migration research

The rapid growth of global international migration has prompted researchers to rethink traditional approaches to the study of migration processes and patterns. Consequently, the transnational approach has been proposed as a comprehensive perspective that focuses on the complex network of relationships and interactions maintained by migrants across national borders.

First systematized in the 1990s by American scholars Glick Schiller et al. (1995), the transnational approach created a conceptual foundation for diverse transnational studies. According to Stepanov (2018), this approach emerged as a response to the crisis of assimilation and multiculturalism theories. In the context of globalization and accelerated international integration, the transnational approach offers a novel perspective on contemporary migration processes (Stepanov, 2018, p. 119). Key aspects of these studies include migrants' constant involvement in everyday life and activities in both the host society and their country of origin, movements between these countries, and other issues related to cross-border interactions.

The concept of transnationalism implies a migrant's inclusion in a transnational social space that spans two or more nation-states. These spaces and their boundaries, described as “part of an ongoing cultural, social, and societal transformation” (Drüeke et al., 2021, p. 162), shape these changes. Principal markers of transnationalism include transnational identities, regular connections and cross-border movements, involvement in the economic and political activities of these countries, transnational movements and communities, and transnational socio-cultural practices involving the circulation of cultural values, symbols, ideas, and material cultural objects (Faist, 2013, p. 452).

Transnational migration is characterized by migrants' simultaneous belonging to two or more societies, evidenced by regular, sustained online and offline contacts, interactions, and activities (Glick Schiller et al., 1995, p. 47; Erlinghagen, 2021, p. 1342).

The notion of migrants as “transmigrants,” actively engaged with both their country of origin and their country of residence, creating transnational social spaces where their social identities are shaped (Glick Schiller et al., 1995, p. 47), has spurred numerous studies in this area (Erlinghagen, 2021). Studies within the framework of transnationalism generally fall into two complementary directions: transnational parenting, with a focus on transnational motherhood, and transnational kinship, centered around the concept of the transnational family in a broader sense, which encompasses interpersonal and intergenerational relationships (Merla et al., 2021, p. 440). Kinship, characterized by extended familial ties, fosters a web of relationships transcending national borders (Macpherson and Macpherson, 2009, p. 74). The term “transnational family” refers to a family that, despite physical separation for a period, preserves its familial identity by collectively fostering a sense of unity and wellbeing (Bryceson and Vuorela, 2002, p. 18). As highlighted by Merla et al. (2021), this concept originated in European science, though its roots in scientific inquiry into transnational motherhood were laid by American scholars. Transnational families, formed as a result of family members migrating to other countries in pursuit of educational, professional, or political opportunities, remain dynamic systems comprising individuals with shared goals and enduring commitments to one another. Additionally, the transnational family serves as a primary mechanism for preserving indigenous culture and heritage language, adeptly navigating both societal norms and cultural heritage (Zabrodskaja et al., 2023, p. 2).

The contemporary transnational family encompasses diverse configurations that transcend both familial and national boundaries. It functions as “a network of interdependent kin and non-kin relationships, facilitating the mobilization and exchange of social resources within and across national borders, in accordance with the family's needs and norms. Furthermore, the family network and resource circulation evolve over the life course” (Nedelcu et al., 2023, p. 4). Consequently, the transnational family, through its adaptive arrangements and maintenance of ties within transnational social spaces and diverse ethno-cultural contexts, undergoes continual transformation manifesting in various configurations. However, Ngan and Chan (2022) differentiate between transnational families with opportunities for their children to study abroad and those of migrant workers. They assert that the separation experienced by migrant workers' families is a forced strategy for economic survival, whereas for higher-income families, the transnational family strategy aims to “enhance social, cultural, and symbolic capital, thereby perpetuating social status and family mobility” (Ngan and Chan, 2022, p. 199). Within this framework, transnational migration is considered a fundamental aspect of family wellbeing, offering distinctive experiences, valuable knowledge, skills, and connections. The pursuit of collective wellbeing and unity is facilitated through various forms and degrees of co-presence (virtual, proxy, physical, and imagined), enabling the management of emotions associated with cross-border relationships, including feelings of absence, longing for individuals and places, and guilt (Baldassar, 2008, p. 248, 250).

Meanwhile, Merla et al. (2021) highlight the extensive thematic representation of transnational kinship/transnational family, emphasizing “the key role of care in maintaining family relationships at a distance” (p. 440). Sampatio and Garvalho (2022), examining migrants' relationships with close relatives concerning caring for elderly parents remotely, note the emotional involvement characteristic of transnational families, characterized by emotional support and a “constant desire to be available”. Mutual care (physical, financial, emotional, moral, and virtual) within transnational families is regarded as a component of wellbeing, constituting a social process encompassing material, subjective, and relational dimensions. It is also influenced by political, economic, social, and migration transformations, which can occur suddenly, such as changes in migration and visa policies (Sampatio and Garvalho, 2022, p. 2).

Baldassar (2008) contends that emotional and moral support, or emotional care, forms the foundation of transnational family relations. The use of modern communication technologies facilitates a sense of simultaneous presence in two locations: the host country and the country of origin. Despite the significant advancements in information and communication technologies, spatial distance continues to be significant in family relationships, particularly for members of different generations separated by distance (Christensen, 2020). Furthermore, time plays a crucial role, encompassing the waiting period for family reunification (Gustafsson, 2022).

The emotional aspect of transnational communication with family and relatives can partly compensate for their physical absence. Badiei and Popkova (2023) argue that the capabilities of communication technologies to express emotions realistically influence individuals' choice of communication methods when communicating remotely. Conversely, the preference for social media platforms with limited emotional expression capabilities is driven by the desire to conceal emotions (Badiei and Popkova, 2023). Matei and Bobârnat (2021) study on “Parental Role Changes in Romanian Transnational Families: Consequences of Migration” reveals profound consequences of parents' migration abroad for the emotional experiences of family members, especially children.

The significance of emotional connections to family becomes especially evident in contexts where there are cultural differences between the receiving and sending societies. To exemplify this notion, two studies, by Shi (2024) and Harris et al. (2020), introduce an intriguing methodology for examining transnationalism through a multidimensional approach, focusing on language. Harris et al. (2020) note that upon their initial arrival, young migrants often rely on family ties from their home country and ethnic connections in the host society. However, as time progresses, they develop broader social relationships across ethnic groups as part of their integration into the host society (Harris et al., 2020, p. 3). In the 2024 publication “Discursive Formation of Personalities: Life Trajectories of a Transnational Doctoral Student between the UK and China”, Yu Shi identifies a significant barrier to the integration of a Chinese-origin respondent into the British academic context, despite his prolonged residence and studies in the UK. This barrier is attributed to differing perceptions of culture between the respondent, raised in the “Confucius cultural zone” valuing modesty and group obedience, and “Americanized” Britons (Shi, 2024). Therefore, “transnational caring practices and processes are mediated by the possibility of sharing care, cultural perceptions of responsibilities, and negotiated obligations within families” (Baldassar, 2008, p. 24).

Scholars note that collectivist cultures, such as Kazakhstani culture, inherently foster a high degree of family closeness (see Badiei and Popkova, 2023, p. 286). Kazakhstani researchers highlight the historical significance of close kinship relations within Kazakh traditional society, emphasizing its foundation on the model of a single family and adherence to tradition, which held almost sacred authority (Abdildin, 2001; Barlybayeva, 2018).

As a result, the transnational approach recognizes the coexistence of diverse identities and attachments stemming from the regular involvement of migrant family members in transnational practices, including interactions across national borders. This approach prioritizes maintaining ties with the country of origin while engaging in various activities in both the host country and the country of origin. Migrants often maintain emotional attachments to their birth country, and the observed mutual care between migrants and their families becomes a crucial characteristic of transnational relations, influencing their emotional, psychological wellbeing, and social adaptation in a new society. This approach underscores the significance of these ties in the overall integration process.

### 2.2 Historical overview of migrations from China and Mongolia

Repatriates from China and Mongolia are ethnic Kazakhs with ancestral roots in Kazakhstan who identify themselves as part of Kazakh culture. Most of these Kazakhs are descendants of those Kazakh families who fled the country in the early 20th century for fear of collectivization, forced expropriation of property, and famine (Kuşçu Bonnenfant, 2012, p. 33; Oka, 2013, p. 4). About 5 million ethnic Kazakhs were living outside Kazakhstan, scattered in more than 40 countries of the world, with the largest number in Uzbekistan (1.5 million), China (1.5 million), Russia (1 million), Turkmenistan (100,000), Mongolia (130,000), Kyrgyzstan (45,000), Turkey (10,000), Afghanistan (440,000), and Iran (15,000) (UNDP, 2006; Kuşçu Bonnenfant, 2008).

That is, after gaining independence in 1991, the Kazakh government issued a call to ethnic Kazakhs residing abroad, urging them to return to their ancestral homeland. Simultaneously, the government anticipated that this repatriation effort would address the significant outflow of Russian labor and revitalize the national culture and language, both of which had suffered under the policy of Russification during the Tsarist and Soviet eras (Kuşçu Bonnenfant, 2008, p. 39–40). This migration formed a crucial component of a broader strategy aimed at reinforcing the country's ethnic and cultural identity and bolstering the Kazakh diaspora, both domestically and internationally. To facilitate integration, the government implemented a series of laws and established new programs focused on national repatriation, adaptation, and integration of ethnic Kazakh migrants. Additionally, specialized scientific centers were created to support these initiatives. By reevaluating the Soviet era, Kazakhstan seeks to understand the underlying reasons for the departure and non-return of numerous families, acknowledging the multitude of factors that influenced their decisions (Kalysh and Kasymova, 2013, p. 15).

Since gaining independence, the repatriation process has unfolded in three distinct stages, each geared toward facilitating the return of Kazakhs and enhancing their adaptation upon resettlement.

The first stage, spanning from 1991 to the early 2000s, marked the initiation of comprehensive repatriation efforts. It was during this period that the term “oralman”, meaning “returnee” in Kazakh, was officially defined in the Law “On Population Migration” dated 13 December 1997. This legal framework delineated oralmans as individuals of Kazakh nationality who were residing outside Kazakhstan's borders at the time of its sovereignty acquisition and subsequently relocated to establish permanent residency within the country (Law of the Republic of Kazakhstan, 1997). Statistics from the Ministry of Labour and Social Protection of the Population reveal that between 1991 and 2000, a total of 42,387 families, comprising 183,652 individuals, migrated to Kazakhstan, constituting over 17% of the total resettled population (Documents and Statistics, 2020).

The second stage, spanning from 2001 to 2011, witnessed a steady rise in repatriation figures. A pivotal document during this period was the “On the Concept of Migration Policy of the Republic of Kazakhstan for 2007-2015” (2007),^1^ which underscored the significance of ethnic Kazakh returnees in bolstering the country's population and fostering natural demographic growth. According to data from the Ministry of Labour and Social Protection of the Population, between 2001 and 2011, a staggering 697,769 ethnic Kazakhs returned to Kazakhstan, constituting over 65% of the total repatriated population since independence (zakon.kz, 2023).^2^

The third and current stage, spanning from 2011 to the present, is marked by the implementation of the Concept of Migration Policy for 2017–2021 (“On Approval of the Migration Policy Concept of the Republic of Kazakhstan for 2017-2021 and the Action Plan for the Implementation of the Migration Policy Concept of the Republic of Kazakhstan for 2017-2021”, 2017).^3^ This policy emphasizes Kazakhstan's commitment to long-term, permanent migration solutions for ethnic repatriates. It prioritizes the creation of conducive environments and incentives for national consolidation, encourages the return of ethnic Kazakhs residing abroad to their ancestral homeland, and aims to facilitate their resettlement in regions experiencing labor shortages. Furthermore, it emphasizes providing support for their adaptation and integration into Kazakh society. Throughout this period, a pivotal development was the official replacement of the term “oralman” with “qandas”.

According to the revised legislation (Law of the Republic of Kazakhstan, 2020), a qandas is defined as an ethnic Kazakh or members of their family who previously lacked Kazakhstani citizenship but have since obtained it through the established procedures by the migration authorities. Furthermore, the term encompasses children of ethnic Kazakhs born and residing outside Kazakhstan after its acquisition of sovereignty (Law of the Republic of Kazakhstan, 2020).

Presently, Kazakhstan's migration policy, outlined in the Concept of Migration Policy for 2023–2027, prioritizes the preservation of national traditions and strengthening economic ties with the historical homeland by engaging ethnic Kazakhs from other countries in Kazakhstan's development (“On Approval of the Concept of Migration Policy of the Republic of Kazakhstan for 2023-2027”, 2022).^4^ According to the Ministry of Social Protection and Labor, a total of 1,123,277 ethnic Kazakhs have returned to Kazakhstan since 1991 (Documents and Statistics, 2020).

The experience of ethnic Kazakhs in different environments intensifies the process of ethnic self-identification while necessitating continuous adaptation to new cultural norms and traditions. However, their return does not result in a homogeneous community due to diverse origins, socio-economic resources, and social communication systems. Researchers note that Kazakh ethnic migrants represent one of the most vulnerable social groups in Kazakhstan, with varying levels of socio-economic integration (Diener, 2005a,b; Finke, 2013). While some repatriates successfully adapt to life in Kazakhstan, others face significant challenges throughout the adaptation process.

Researchers highlight that repatriates from Mongolia and China exhibit exceptional organization, particularly in their preparation for relocation. They actively mobilize support networks, utilizing social media platforms to coordinate assistance from family members, friends, and fellow tribesmen. Moreover, they demonstrate a proactive approach in establishing businesses and engaging in various forms of social interaction, such as compatriotship (Kalysh and Kasymova, 2013; Bokayev et al., 2014). Specifically, Kazakhs from China are noted for their resilience, having undergone rigorous political education within the framework of the constructed autonomy and competing identity systems in the Xinjiang Uygur Autonomous Region (Kalysh and Kasymova, 2013, p. 174–175). This immersion in China's educational structure equips young repatriates with valuable skills, potentially enhancing their preparedness for higher education in Kazakhstan.

It is essential to recognize that each successive wave of ethnic Kazakh arrivals in Kazakhstan arrives with prior knowledge of the challenges ahead, adopting more efficient coping strategies. Typically, younger family members lead the migration, while the older generation provides emotional and moral support (Bokayev et al., 2014; Taldybayeva et al., 2021).

Ethnic Kazakh students, despite their upbringing in a different country, possess a strong sense of national identity, aspirations, and shared values characteristic of Kazakhs. Their commitment to embracing Kazakhstan, mastering the Kazakh language, and preserving the country's territorial integrity is evident in their interactions. However, upon arrival, they encounter various challenges, not only within the social environment but also within the educational system (Zharkynbekova and Bokayev, 2011).

Crucially, their dual cultural and linguistic heritage—resulting from historical, cultural, and linguistic factors—shapes their mentality, worldview, lifestyle, and language. As bilingual individuals, they navigate the dynamic interplay between Chinese/Mongolian and Kazakh languages, as well as the convergence of Chinese/Mongolian and Kazakh cultures, which profoundly influences their adaptation process (Bokayev et al., 2014; Kalysh and Kasymova, 2014; Mendikulova, 2006).

## 3 Materials and methods

### 3.1 Educational policy of the Republic of Kazakhstan in relation to students—ethnic Kazakh migrants

Young ethnic Kazakhs encounter diverse challenges and opportunities in contemporary society, where their interaction with modern culture, preservation of traditions and language, and adaptation to new social norms significantly influence their psychological wellbeing. Educational policy aimed at the younger generation of repatriates is crucial for their successful integration into society. Notably, the older generation of ethnic Kazakhs, motivated by the desire to provide favorable conditions for their children's development in a native cultural environment and facilitate further social advancement, emphasizes the importance of obtaining necessary professional skills and higher education qualifications as essential prerequisites for employment.

The government of Kazakhstan views education as a key mechanism for leveling opportunities in the socio-economic sphere, particularly for those with unequal starting points. To achieve this goal, the government is actively working to ensure that young repatriates and foreign ethnic Kazakhs have access to higher education. A significant step toward this objective was the Resolution of the Cabinet of Ministers of the Republic of Kazakhstan dated September 23, 1992, No. 791, which allocates special state quotas for this category of citizens upon admission (The Resolution of the Cabinet of Ministers of the Republic of Kazakhstan, 2006).

Since 2007, preparatory departments have been established in higher educational institutions to equip students with the necessary skills for university admission. Currently, there are 14 such departments across various universities. For instance, in 2021, L.N. Gumilyov Eurasian National University enrolled 615 individuals, with 156 from China and 459 from Mongolia. Similarly, in 2022 and 2023, enrollment figures remained substantial, indicating ongoing efforts to facilitate access to higher education for repatriates and foreign ethnic Kazakhs.

**Figure 1:**
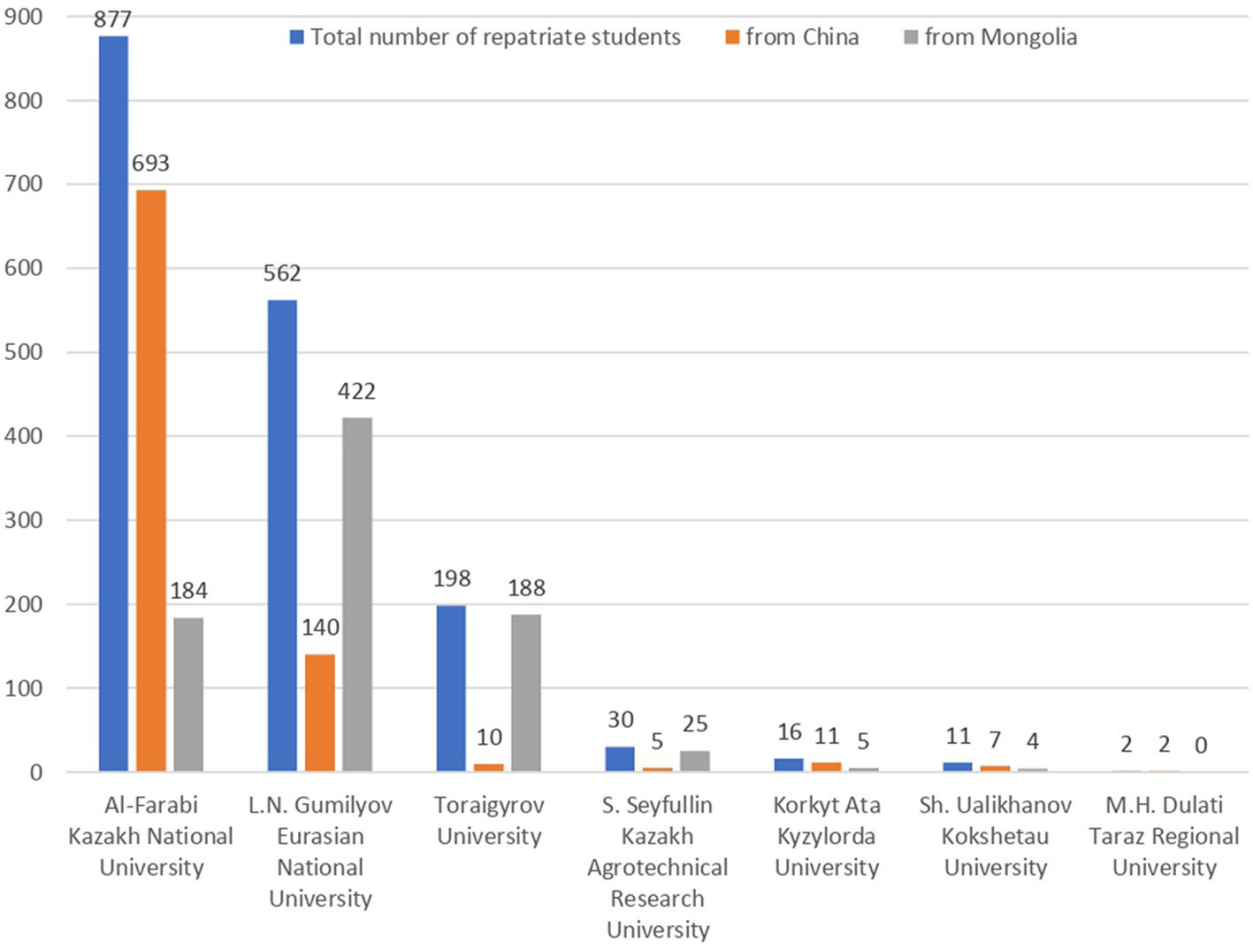
Representation of repatriate students in the Kazakhstani universities under consideration.

The total number of repatriate students from China and Mongolia in the Kazakhstani universities under consideration is shown in Figure 1.

Education operates as an open system, fostering communication between society and its challenges and young people through knowledge interpretation. It enables young repatriates to reframe their understanding of the past, assess present inequalities as temporary, and lay the groundwork for a shared future through a controlled competitive educational process (Kalysh and Kasymova, 2013, p. 175). Integration into the socio-cultural and educational fabric of modern Kazakhstan can be facilitated through the education process, promoting differentiated value attitudes, ethical standards, and communal living practices. This encompasses adherence to sanitary and hygiene standards, as well as the evaluation of value motivations and judgments.

Our long-term observations of the communication behavior of students from China and Mongolia have allowed us to identify some of their distinct characteristics, including a special tolerance, restraint, emphasis on respect for elders and the environment, a certain level of secrecy, and a desire to maintain close contact with compatriots from their countries of origin (Zharkynbekova and Bokayev, 2011). There is also a different degree of adaptation of students who arrived from Mongolia and China. Students from Mongolia are close to the cultural environment of the post-Soviet space as they demonstrate a quicker adaptation to the changes in Kazakhstan and a more facile acquisition of writing skills compared to their counterparts from China. This discrepancy can be attributed to several factors. In China, the education system primarily employs hieroglyphic writing, leaving young individuals unfamiliar with the Cyrillic alphabet used in Kazakhstan. Additionally, differences in political attitudes, ideology, and cultural norms between the two countries contribute to the challenges faced by Chinese students upon their arrival in Kazakhstan. For instance, findings from a survey conducted by the authors of this article highlight that a significant challenge encountered by students upon entering the educational environment is their lack of proficiency in the Russian language. Kazakhstan's linguistic landscape is characterized by ethnic bilingualism, which stems from its historically diverse population composition. The interaction between Kazakh and Russian languages spans centuries, marked by profound mutual influence driven by various cultural-historical, political, socio-demographic, geographical, economic, and linguistic factors. Despite ongoing shifts in the ethno-linguistic dynamics and the redistribution of language usage within Kazakhstan's communicative sphere, Russian remains the predominant language of interethnic communication, spoken by the majority of the population (Zharkynbekova and Chernyavskaya, 2022a,b). Nevertheless, while significant changes are evident in the overall linguistic landscape, the situation remains predominantly centered around Kazakh and Russian languages, as noted by Suleimenova (2010, p. 27).

At the same time, Kazakh students from China experience greater language difficulties than students from Mongolia, who claim that they can read and understand what is written in Russian or Kazakh. For ethnic Kazakh students from China, this takes some time. Cultural differences between newcomers and local people also play a role, of course. As noted by Tabea and Finke (2013), some repatriates of the 1990s after 2–3 years of living in Kazakhstan, returned to the country where they came from, unable to get used to the local environment and climatic conditions.

### 3.2 Research tools

The study relied on a comprehensive array of sources, including official statistical data from state bodies of the Republic of Kazakhstan such as the Bureau of National Statistics and the Ministry of Labor and Social Protection of the Population. Normative legal acts, including the Constitution of the Republic of Kazakhstan and the Law “On Population Migration” dated July 22, 2011, along with policy documents like the Concept of Migration Policy for 2017-2021 (2017) and 2023-2027 (2022), were also pivotal to the research. Additionally, periodical materials capturing the current state of affairs and internet sources, notably the websites of the Chinese and Mongolian Embassies in Astana, Republic of Kazakhstan, were consulted. Furthermore, the study engaged with existing research by domestic and foreign scholars dedicated to the pertinent issue.

The methodology employed by the authors encompassed both surveying and conducting interviews with students from China and Mongolia, all conducted in their native Kazakh language. The questionnaire, developed based on several key indicators, addressed cognitive, axiological, and behavioral aspects of cultural diffusion. These indicators encompassed attitudes toward the values and norms of the host community, family relations including the importance of parental attention, behavioral patterns and interactions with the host society, cognitive aspects such as interest in other cultures and willingness to engage with them, and openness to accepting certain aspects of other cultures. The survey was conducted in 2023, with a sample comprising 530 respondents aged 18–25, enrolled in national and regional universities across five regions.

The universities included in the study were S. Seyfullin Kazakh Agrotechnical Research University, Toraigyrov University, Korkyt Ata Kyzylorda University, Sh. Ualikhanov Kokshetau University, and M.Kh. Dulati Taraz Regional University, representing the regional institutions, while Al-Farabi Kazakh National University and L.N. Gumilyov Eurasian National University represented the national level. During the interpretation phase, correlations were drawn with regards to gender, age, ethnicity, level of education, and socio-professional affiliation, providing a nuanced understanding of the data.

The questionnaire comprised three distinct blocks, each serving different investigative purposes. The first block focused on elucidating the socio-economic characteristics of the respondents, including their social status, country of arrival, age, gender, and duration of residence in the host country (refer to Table 1).

**Table 1:** Distribution of informants by country of stay, age, gender, time of residence in the country of arrival.

**Country of origin**	**Age of respondents**		**Gender**		**Length of stay in Kazakhstan**		
	**18–20 years old**	**21–25 years old**	**Male**	**Female**	<**1 year**	**From 1 to 3 years**	**From 3 to 5 years**
China	83	67	61	89	84	53	13
Mongolia	212	168	217	163	196	148	36

In contrast, the second block aimed to assess the participants' adaptation to the new culture and society, identifying factors influencing the adaptation process and pinpointing areas requiring additional support. Meanwhile, the third block delved into the emotional and interpersonal dimensions of family relationships, the preservation of cultural traditions, and the respondents' aspirations for the future.

The study design incorporated a qualitative methodology known as biographical interviews, guided by a predefined list of key topics. Employing discourse analysis, the researchers scrutinized the interview texts to discern various discursive strategies, themes, and ideas. It is important to note that this methodological approach does not purport to generalize its findings to the entire ethnic Kazakh population from China and Mongolia. The interview topics spanned diverse aspects of life, encompassing childhood experiences, educational trajectories, family dynamics, migration experiences, and future plans, particularly for those who relocated to Kazakhstan and acquired citizenship.

Between September 05, 2023, and December 30, 2023, a total of 36 interviews were conducted. Respondent selection employed a non-random sampling method, combining purposive and spontaneous approaches. The organizers' high level of trustworthiness facilitated in-depth interviews, allowing for a rich exploration of the respondents' life narratives. Notably, the participants consisted of 36 individuals aged between 18 and 25, comprising 16 males and 20 females.

Based on the collected data, the researchers categorized all informants by gender, country of origin, and length of stay in Kazakhstan (see Table 2).

**Table 2:** Distribution of interview informants by country of arrival, length of stay and gender.

**Country of origin**	**Age of the respondents**		**Gender**		**Length of stay in Kazakhstan**		
	**18–20 years**	**21–25 years**	**Male**	**Female**	<**1 year**	**1–3 years**	**From 3 to 5 years**
China	8	6	5	9	7	5	2
Mongolia	12	10	9	13	9	7	6

The categorization by length of stay, specifically distinguishing between stays of up to a year and three or more years, was motivated by several factors. Firstly, the duration of residence in a new social milieu directly influences the repatriate level of adaptation and socio-psychological resilience. Secondly, the unique living and socialization conditions within Kazakhstani educational settings were considered, with young ethnic Kazakhs typically spending their first year in preparatory departments alongside similar peers before integrating into mainstream courses alongside local students of various backgrounds. Lastly, researchers suggest that a stay of three or more years provides sufficient time to observe notable shifts in migrants' identity, culture, and language from their country of origin (Khilkhanova and Khilkhanov, 2020, p. 32).

Informal interviews were conducted with representatives of the target group, comprising students who migrated to Kazakhstan for educational purposes. These interviews were recorded using DVF format software and subsequently transcribed for analysis. In examining the survey findings, standard methods of mathematical statistics were employed, including procedures for standardizing scales, Cronbach's alpha coefficient, Student's *t*-test, and correlation analyses using Spearman's rank correlation coefficient (*R*) and Pearson's correlation coefficient (*p*). Data analysis was conducted using the statistical software package SPSS 27.0.

The authors of the study drew upon their extensive pedagogical experience working with Kazakh youth from China and Mongolia to provide additional insights into the survey results. These insights were based on their observations of the daily lives of repatriate students both within and outside the university environment.

We believe that the combination of quantitative and qualitative approaches, coupled with the authors' pedagogical insights, offers a comprehensive understanding of the perspectives of young ethnic Kazakhs from Mongolia and China studying in Kazakhstan. This methodological approach, aligned with contemporary sociolinguistic priorities, facilitates a nuanced exploration of the research questions at hand, taking into account both objective and subjective assessments of the participants' experiences.

## 4 Results and discussion

### 4.1 Main empirical findings: transnational practices

Within the framework of this 41-point questionnaire project, 145 respondents were interviewed, which allowed us to collect a significant amount of data on demographic characteristics, language practices, length of stay in Kazakhstan and motives for moving, connections with relatives, etc.

The questionnaire questions also covered aspects of adaptation to the new socio-cultural environment, including the perception of cultural dualism, the availability of social support and the impact of cultural differences on personal and family relationships. The respondents shared their experience in managing the emotional and psychological aspects of separation from family, as well as strategies for maintaining long-distance relationships.

The study paid special attention to the role of family support in the process of adaptation, the preservation of cultural traditions and the use of various means of communication to maintain contacts.

The internal consistency scale is presented in Table 3.

**Table 3:** Cronbach's alpha reliability statistics.

**Cronbach's alpha**	***N* elements**
0.762	41

The Cronbach's Alpha coefficient is a measure of the internal consistency of a scale used to measure the same concept among observed variables. A value of 762 indicates satisfactory reliability of the scale (refer to Table 3).

Further, the following statistical parameters were calculated for each of the questions: minimum and maximum values, average value, standard deviation and variance.

The minimum and maximum values varied depending on the number of possible answers to each question, where the minimum value corresponded to the smallest answer option, and the maximum value corresponded to the largest.

The average values indicated a general trend in responses. For example, for the question about the attitude to adaptation in a new cultural environment, the average value was 2.0690, which may indicate a rather positive trend in the perception of adaptation among respondents.

The standard deviations and variances showed the degree of variation of responses relative to the average value. Higher values of these parameters indicate a greater variety of responses among respondents. For example, the question about the difficulties faced by the participants in Kazakhstan has a variance of 0.946, which indicates a wide range of experiences among the respondents (see Table 4).

**Table 4:** Sample items from questionnaire.

**Questionnaire item**	**Minimum**	**Maximum**	**Average value**	**Standard deviation**	**Variance**
What language do you speak at home?	1.00	3.00	1.3241	0.65493	0.429
Does your family live in Kazakhstan?	1.00	4.00	2.5517	0.56435	0.318
How long have you been living in Kazakhstan?	1.00	2.00	1.8621	0.34602	0.120
What are the main reasons for your coming to Kazakhstan?	1.00	4.00	2.1655	0.84995	0.722
How was the adaptation process in Kazakhstan?	1.00	4.00	3.1724	1.20960	1.463
How do you feel about adapting to a new cultural environment?	1.00	4.00	2.0690	0.67346	0.454
What do you believe to be the key factor in your adaptation to Kazakhstani context?	1.00	4.00	1.8069	0.61573	0.379
What difficulties did you encounter in Kazakhstan (main problem)?	1.00	4.00	1.8897	0.97266	0.946
Have you experienced pressure or faced negative attitudes because you are from another country?	1.00	4.00	2.2897	0.76301	0.582
Has your time here affected your family relationships?	1.00	4.00	2.4000	1.00277	1.006
How often do you communicate with your family?	1.00	4.00	2.1793	1.01152	1.023
How does your family support you in dealing with problems or difficulties?	1.00	3.00	1.4966	0.67828	0.460

The indicator of the language used at home, with an average value of 1.3241, and a relatively high standard deviation, indicates a variety of language practices, which reflects the diversity of the cultural background of students. This diversity, in turn, can influence how students communicate within the family and perceive the integration process in a new country.

The mean score for the reasons behind migrating to Kazakhstan is 2.1655, indicating a considerable variance. This underscores the diverse motivations and circumstances behind each individual's decision to relocate, with each case presenting unique challenges for adaptation and family dynamics. Furthermore, the mean score for the adaptation process is 3.1724, indicating a relatively high level of adaptation overall. However, the significant standard deviation and variance highlight the wide range of personal experiences students have had with adaptation. Consequently, while some individuals may feel well-adjusted, others may encounter substantial difficulties in their transition.

Regarding family relations, it is important to note that the average value of the question of physical intimacy with family is 2.6690, which may indicate the importance of family intimacy and its impact on emotional wellbeing, confirmed also by a significant standard deviation in this issue. Statistics on the family's attitude to the decision to migrate for study, with a low average of 1.6759, indicate a tendency for families to support such decisions, which can be interpreted as a sign of strong family foundations and support.

Subsequently, we conducted a correlation analysis to identify the most significant relationships. The Pearson correlation coefficient (r) was employed to quantify the strength of the linear relationship between two quantitative variables. This coefficient ranges from −1 to 1, where a value of 1 denotes a positive linear correlation, −1 indicates a negative linear correlation, and 0 signifies no linear relationship. Coefficient values approaching 1 or −1 indicate a robust correlation, while values nearing 0 suggest a weak correlation.

To interpret the Pearson coefficient, the following should be considered:

- *Values* > *0.5 or* < –*0.5 are usually interpreted as indicating a moderate to strong correlation*.
- *Values from* –*0.5 to 0.5 can be considered a weak correlation*.
- *Values close to 0 mean that there is no linear correlation*.
- *Values closer to 1 or* –*1 indicate a stronger linear relationship between the variables*.

Attention should be paid to the coefficient values, which are statistically significant and have a large absolute value, as they indicate a more significant correlation. For accurate interpretation, it is also important to consider the *p*-value, which allows us to judge the statistical significance of the detected correlation. A *P*-value < 0.05 is usually considered an indication of a statistically significant correlation.

To address the research questions posed in this article, several key issues are highlighted. Specifically, correlations between family relationships among students and their parents in China and Mongolia (questions 17–21, 27, 29, 30) are examined to explore how these dynamics influence their adaptation in Kazakhstan. Additionally, questions related to adaptation and integration into a new cultural environment (e.g., questions 9–14, 20, 34) are pertinent to understanding the impact of family relationships on the adaptation process of ethnic Kazakh students from China and Mongolia.

By analyzing significant correlations within the research and aligning them with the research questions, interpretations can be drawn as follows:

Question 17: “Has your stay here affected family relations?”

- Correlation, 209^*^ with question 16 (“Which aspects of local culture were the most difficult for you?”) It may indicate that adaptation to local culture affects family relationships. The more complex aspects of culture are, the greater the impact they can have on family relationships.
- The correlation, 214^**^ with question 20 (“Does family connection help you adapt better?”) suggests that close family connection can help mitigate the impact of changes in family relationships caused by relocation.
- Correlation, 171^*^ with question 31 (“What cultural traditions of your family do you actively support in Kazakhstan?”) emphasizes that maintaining family cultural traditions can strengthen family ties, despite physical absence.

Question 18: “How do you deal with the emotional aspects of physical separation from your family?”

- Strong correlation, 257^**^ with question 13 (“Is it difficult for you to find a balance between your culture and the culture of your country of residence?”) It may indicate that dealing with cultural differences can increase the emotional stress associated with separation from family.
- The correlation, 171^*^ with question 20 (“Does family connection help you adapt better?”) highlights the importance of family support in managing emotional difficulties due to separation.

Question 19: “What difficulties do you face in maintaining a strong family relationship?”

- The correlation, 219^**^ with question 20 (“Does family connection help you adapt better?”) suggests that family connection not only helps you adapt but can also reduce difficulties in maintaining family relationships.
- The correlation, 182^*^ with question 27 (“Is it possible in your family to openly discuss family problems?”) suggests that openness and the possibility of discussing problems in the family makes it easier to maintain strong relationships.

Question 20: “Does connecting with your family help you adapt better?”

- Strong correlation, 264^**^ with question 21 (“How do you deal with the lack of physical intimacy with your family?”) confirms that family connection is critical to overcoming the emotional difficulties associated with physical separation. This indicates that regular communication with family can serve as an effective means of maintaining emotional wellbeing at a distance.

The provided Pearson correlation table shows the relationships between various variables related to adaptation and stay in Kazakhstan. Here are a few key correlations that stand out in particular importance:

In general, there is a significant negative correlation between age and adaptation to a new environment (*r* = −0.299, *p* < 0.001). This may indicate that younger people adapt better to time zone changes compared to older ones.

There is a strong positive correlation between housing conditions and social support (*r* = 0.348, *p* < 0.001), which suggests that the best housing conditions are associated with a high level of social support.

An interesting positive correlation between the participant's gender and the reasons for moving to Kazakhstan (*r* = 0.232, *p* < 0.01) may indicate that men and women may have different motives for migration.

There is a significant positive correlation between the length of stay in Kazakhstan and stability in family relationships (*r* = 0.313, *p* < 0.001). A long stay can contribute to stronger family ties.

A strong positive correlation was observed between cultural adaptation and the presence of friends (*r* = 0.284, *p* < 0.001), which indicates the importance of social ties in the process of adaptation to a new culture.

If we consider correlations in the context of a research question, the following connections seem to be the most significant:

*r* = 0.356, *p*-value = 0 in the question of *adaptation to a new culture and cultural traditions of the country of origin* indicates a strong connection between adaptation to a new culture and the preservation of cultural traditions of the country of origin, which can play an important role in the adaptation of students.

In the question *of frequent communication r* = 0.311, *p*-value = 0 shows that frequent communication can significantly affect the processes of adaptation and integration.

The correlation between students' age and adaptation experience also shows a positive value (*r* = 0.348, *p*-value = 0).

The inverse correlation *of the adaptation process and open discussion* may mean that difficulties in the adaptation process may be associated with a lack of open discussion of problems (*r* = −0.309, *p*-value = 0). It is also worth emphasizing that the correlation between the questions *sharing difficulties with loved ones and open discussion r* = 0.417, *p*-value = 0: The high correlation between these two variables emphasizes that the opportunity to discuss their problems with loved ones helps to improve emotional state and promotes adaptation. This confirms the need to maintain strong family ties and provide support to students during transition periods.

Correlation = 0.326, *p*-value = 0 in the question of *aspects of a new life* indicates the importance of the influence of various aspects of a new life on the adaptation process.

*Cultural traditions of the country of origin* (*r* = 0.393, *p*-value = 0) shows a strong connection between the preservation of cultural traditions and successful adaptation, which indicates the importance of respect for the cultural baggage of students in the process of their integration into a new social and educational environment.

A biographical interview was conducted as an additional research tool. Discourse analysis of the interview texts made it possible to identify various themes, opinions, ideas, and experiences expressed in them. This approach allowed us to better understand how respondents construct their arguments, form their positions, express emotions, and convey their feelings.

### 4.2 Discourse interpretation of repatriate students' narratives

#### 4.2.1 The role of family in the lives of qandas students from China and Mongolia

Discourse analysis of student essays confirms that family plays a dominant role in the lives of qandas students. The most significant for qandas students are such traditional values as “family” (70.5%), “religion” (47.4%), “respect for parents and elders” (38.6%), “independence of Kazakhstan” (36.8%), “responsibility to one's clan/tribe” (32.9%), “responsibilities/duty to the family” (31.2%), which indicate the importance of the family in determining their future related to their ethnic homeland (motivational goals). Nevertheless, despite the rather strong emotional attachment to the country of origin, most respondents plan their future life in Kazakhstan, since obtaining higher education in Kazakhstan meets the expectations and hopes of parents to return and return their heirs to their historical homeland, and each of the respondents feels their responsibility to the family: “*It was difficult for me since I am the youngest in the family. But I came here to study in order* ***to meet the expectations of parents*** *and I decided to continue my studies*” (Zhuldyz, 19 years old, from Mongolia); “*My parents were also born and raised in Mongolia. They also wanted to return* ***to their homeland***. ***I hope that in the future they*** ***will also*** *move here*” (Meruert, 19 years old, from Mongolia); “*I will stay* ***here**. I will become a citizen* ***before graduating from university***, ***there is no turning*** ***back***” (Rita, 20 years old, from China). The possibilities of personal growth and professional development in the homeland of ancestors are an undoubted argument in considering the future in Kazakhstan: “*My changes before and after arriving are my development* ***in Kazakhstan*** *are my development. Because there are differences in the political and cultural development of Mongolia and Kazakhstan. Therefore*, ***I imagine*** ***my future in Kazakhstan***” (Dauren, 20 years old, from Mongolia); “*My aim is to study in Kazakhstan* ***and become a good specialist***” (Tulpar, 19 years old, from Mongolia).

The categories “impeccable reputation” (36.9%), “hard work” (36.3%), and “good education” (36.2%), as presented in the responses of respondents, can be considered markers defining the values embedded in the family. In the traditional Kazakh family, it has been considered important to bring up children as worthy citizens for centuries. Furthermore, respondents identified the importance of wealth and money (34.4%), freedom of choice (31.8%), and equality/equal rights (30.7%) in modern life.

#### 4.2.2 Language practices in qandas families

The upbringing of family values and language policy is widely acknowledged to be shaped by “ideology, management, and practice” (Seppik and Zabrodskaja, 2022), influenced by a complex interplay of “internal (language management strategies within the family, implicit and explicit language choices, attitudes and efforts to maintain and transmit the heritage language, affective sphere, the role of child agency, and communication with siblings) and external (socio-economic status of the family, welfare, social network, collaboration with educational institutions, interaction with teachers and experts, quality and quantity of materials for the heritage language, and societal language) factors” (Zabrodskaja et al., 2023). Following this concept, it is possible to explain the nature of communication in transnational qandas families. The younger generation of qandases communicates with the older generation in their native Kazakh language, and in communication with peers and siblings, along with their native language, they choose the language of society (Chinese/Mongolian): “*We communicate with friends from the preparatory group in Chinese. They are also Kazakhs who returned from China”* (Rita, 20 years old, from China). So, if the choice of mother tongue is dictated primarily by parental expectations, attitudes and efforts to maintain and transmit the language of heritage (internal factors), then the choice of the language of society is determined by dominant interaction with the environment (external factors). Accordingly, the main difficulty in adapting qandas students from China and Mongolia in Kazakhstani society was presented by the lack of mastery of the Russian language—the “language of society” (Zabrodskaja et al., 2023) as a necessary condition for development and prosperity in this multinational society: “*When I first came* ***it was very difficult*** *because I couldn't speak Kazakh properly and the teachers also spoke Russian, so* ***I didn't understand***. ***My classmates were teasing me with*** **‘*oralman'***. *Then I was crying. I didn't know Kazakh very well then. After I learned the language, I began to understand what they were talking about. But there were good classmates who helped me with everything” (Salamat, 18 years old, from China); “**I am afraid of Russian***, ***don't understand at all**. Even though I'm trying to learn it*, ***I just can't seem to get it**. If I'm standing at a bus stop and there's a* ***Russian next to me***, ***I immediately try to distance myself**, so that he doesn't ask me questions' (Salamat, 18, from China)*; *due to the fact*, ***that many people here*** ***speak Russian**, when we arrived*, ***we did not know the language**. These were the difficulties. When we were at school, we only worked with a dictionary, we did not learn spoken language, and when we came here, we slowly learned spoken language. That's why we know the words, but we don't know how to use them.” (Dosbolat, 21 years old, from Mongolia)*. At the same time, difficulties also arose with the “heritage language” (Zabrodskaja et al., 2023): “*I studied purely in Chinese so* ***understanding Kazakh was very*** *difficult* ***for me***. ***I didn't learn Kazakh letters*** *so* ***quick reading***, ***correct speech***, ***and writing*** *is still a problem* ***for me***”* (Buldirshin, 18, from China); “It was a little difficult at school because in China we studied in Chinese* ***didn't know letters*** *because there are differences in the Kazakh language and because we have not learned Russian before*, ***we didn't understand Russian*** *then there were difficulties with the Russian language” (Bayan, 18 years old, from China)*.

Despite being fluent in spoken Kazakh from birth, qandas students faced challenges studying in the heritage language due to difficulties with writing and terminology.

#### 4.2.3 How do qandas students experience separation from their parents?

According to respondents, maintaining communication with family and friends helped them navigate these challenges, primarily through social media platforms and messaging apps. They relied on domestic media for information and some students even started blogging about travel, local cuisine, and traditions, finding it both a distraction and a source of entertainment.

For successful adaptation, repatriate students must strike a balance between two cultures: Kazakh and Chinese/Mongolian. This duality gives rise to dual identities, shaped by their long-term upbringing in their home country and their current experiences in Kazakhstan.

Zhuldyz, a 19-year-old student from Mongolia, emotionally expressed her newfound appreciation for family amidst separation: “*Actually, it seems to me that family relations have only strengthened because I had not left Ulan-bator for a long time before*, ***you begin to appreciate home, and miss it***. *If I'm happy, I laugh. If I'm sad, I cry. Sometimes, even small things can make me cry. I don't really show my emotions”*.

Students' responses indicate a strong emotional concern for their parents. Nearly all participants expressed their intention not to burden their parents with their own challenges. During video calls or other forms of communication, they tend to share only positive aspects of their experiences while keeping silent about any difficulties they may be facing: “*I'm* ***afraid to tell my parents about my problems**. They cannot help*, ***they will*** *only* ***worry***” (Salamat, 18, from China). “*No, I* ***don't tell*** *them* ***everything***. ***I don't want them to worry***” (Akmaral, 19, from Mongolia). “***I don't tell them (parents)**. Just friends. I* ***don't want my*** ***parents to worry***.” (Zhuldyz, 19, from Mongolia); “*As they are far away, I miss my family. Sometimes I do not know how to calm my sadness. Sometimes there are moments when I cry a lot. However*, ***I try not to let my family know about it***.” (Tulpar, 19, from Mongolia). Recognizing that their parents cannot help them solve their problems due to the distance between them, they consciously protect them from stress. Caring for parents is demonstrated through “selectivity, omission, and silence, which are key elements of emotional care” (Lin Sin and Schartner, 2024). They share their problems with friends, mostly from the country of origin, who live both here and in other cities of Kazakhstan. Moreover, emotional connection with friends, and their support appear to have a positive impact on both the psycho-emotional and academic wellbeing of respondents: “*I have many friends from Mongolia in Astana who came here to study as I did. When we miss our country and relatives* ***we get together***. *We listen to the songs, and music helps us* ***to cope with sadness**. Since we all came here to study, we are* ***like a family**, and* ***are always ready to*** ***help each other***” (Tulpar, 19, from Mongolia); “*Last year, we met students from Mongolia in the preparatory department. There were about 400 students, 100 of them enrolled in the Eurasian National University. We communicate* ***very closely with*** ***each other***, ***and have become friends. We walk together***, ***communicate***, ***and spend*** ***our time together***. *Some of our friends speak English well and others are good at Maths. We all* ***help each other*** *with the lessons*” (Dosbolat, 20 years old, from Mongolia).

It is important to emphasize that modern opportunities provided through communication technologies and social media, as well as the possibility of physical presence (parents staying in Kazakhstan during the child's education, as well as the visa-free regime between Kazakhstan and China/Mongolia, allowing relatives to visit each other), contribute to the psychological wellbeing of repatriate students: “***I went to Mongolia*** *on the* ***weekend**. There (in Mongolia) there are a lot of my friends, and some of them moved to Kazakhstan”* (Dosbolat, 20, from Mongolia). “*We communicate with friends and relatives from China* ***through WeChat several times a month***, ***we make video calls***. *(Rita*, 20 years old, *from China)*”.

However, in the writings of repatriate students, particularly in the initial years, there persists a “feeling of absence and longing for people and places” (Baldassar, 2008, p. 250). Baldassar (2008) elucidates that these emotions, which articulate a “desire to return home”, manifest in several primary ways: through language (discursively), through the body (physically), through actions (practices), and through ideas (imagination) involving all senses: visual, auditory, and more (Baldassar, 2008, p. 250).

Textual analysis reveals that while the longing to *see and hear* is somewhat fulfilled through communication technologies, and the desire for physical *touch* (such as *hugs*) can be satisfied during occasional visits, the yearning for traditional *foods, smells*, and *tastes* associated with their homeland remains unmet. Hence, our respondents miss the authentic atmosphere, Chinese/Mongolian cuisine, and struggle to adapt to or accept local food: “*If I were to be specific*, ***I miss China**, but what could I do, I had to return to Kazakhstan**; there are a lot of dishes in China, a lot of spicy*** ***food**. In Kazakhstan I only like doner”* (Rita, 20 years old, from China). “***I miss the*** ***nature*** *of* ***Mongolia***, ***my childhood***, ***food***, ***my mom and brother”*** (Dosbolat, 20, from Mongolia).

#### 4.2.4 Some differences in adaptation to the new environment of qandas students from China and qandas students from Mongolia

To analyze the correlation and adaptation of students from China and Mongolia, we focused on the frequency and methods of communication with relatives, language preferences, physical distance from family, and attitudes toward returning to their native countries.

**1. Communication and support**: Repatriate students from China and Mongolia demonstrate different aspects of adaptation. According to the data, students from Mongolia are more likely to feel supported through communication with relatives and strive to maintain active contact, which can contribute to more effective adaptation. In contrast, ethnic students from China may experience a greater sense of loneliness due to less frequent visits, although they are more likely to use video calls, which can help mitigate the effects of distance.

**2. Language preferences**: Qandas students from China are more likely to speak the language of their country of residence (Kazakhstan), which may contribute to faster socialization and adaptation. On the other hand, repatriate students from Mongolia, who use the language of their country of origin, may face greater challenges in integrating into the new society.

**3. Physical distance and emotional impact**: The distance between the students and their families significantly impacts their emotional state. Migrant students from Mongolia are more adapt at managing this by employing a range of communication strategies. In contrast, repatriate students from China are more likely to experience feelings of loneliness and longing, which can hinder their ability to adapt.

**4. Future plans and adaptation**: Ethnic Kazakh students from China are more likely to plan to return to their country of origin, which may indicate weaker adaptation or a stronger connection with their homeland. In contrast, repatriate students from Mongolia are more inclined to see their future in Kazakhstan, which may contribute to their better adaptation.

## 5 Conclusion

The integration of repatriate students into Kazakhstani society varies significantly based on external and internal factors such as Kazakhstan's migration policy, duration of residence in the country, economic conditions, climate, level of education, local community attitudes, and individual personal characteristics. The family environment and the strength of family relations are of particular significance within this system. Repatriate students maintain strong ties with relatives and other close contacts in their country of origin, with the majority (82%) continuing to keep in touch after moving to Kazakhstan. Most communicate through social networks, messengers, and telephone calls, while a relatively small proportion visit their country of origin.

The findings indicate that students from Mongolia may adapt more readily to a new country due to more active communication with relatives and a greater willingness to integrate into local society. In contrast, Chinese students, despite utilizing modern technology for communication, experience greater difficulties due to emotional issues related to the lack of physical proximity to their families and frequent plans to return home.

## Data Availability

The original contributions presented in the study are included in the article/Supplementary material, further inquiries can be directed to the corresponding author.

## Footnotes

^1^ https://adilet.zan.kz/rus/docs/U070000399

^2^ https://www.zakon.kz/obshestvo/6408117-skolko-kandasov-vernulis-v-kazakhstan-s-1991-goda

^3^ https://adilet.zan.kz/rus/docs/P1700000602

^4^ https://adilet.zan.kz/rus/docs/P2200000961
