# Supplementary material for: Transnational practices of Kazakh repatriates: the role of family in the adaptation of ethnic Kazakh students from Mongolia and China
Source: Front Sociol. 2024 Sep 13;9:1452785. doi: 10.3389/fsoc.2024.1452785 (PMC11428197; doi:10.3389/fsoc.2024.1452785)
Supplement: Supplementary file 1 [file Table_1.docx]

**Supplementary material**. **Descriptive statistics**

| **Questionnaire items** | **Minimum** | **Maximum** | **Average value** | **Standard deviation** | **Variance** |
| --- | --- | --- | --- | --- | --- |
| How old are you? | 1,00 | 4,00 | 1,0828 | ,46403 | ,215 |
| What's your gender? | 1,00 | 2,00 | 1,4483 | ,49904 | ,249 |
| What country are you from (your place of birth)? | 1,00 | 2,00 | 1,6069 | ,49013 | ,240 |
| What language do you speak at home? | 1,00 | 3,00 | 1,3241 | ,65493 | ,429 |
| Does your family live in Kazakhstan? | 1,00 | 4,00 | 2,5517 | ,56435 | ,318 |
| How long have you been living in Kazakhstan? | 1,00 | 2,00 | 1,8621 | ,34602 | ,120 |
| What are the main reasons for your coming to Kazakhstan? | 1,00 | 4,00 | 2,1655 | ,84995 | ,722 |
| How was the adaptation process in Kazakhstan? | 1,00 | 4,00 | 3,1724 | 1,20960 | 1,463 |
| How do you feel about adapting to a new cultural environment? | 1,00 | 4,00 | 2,0690 | ,67346 | ,454 |
| What do you believe to be the key factor in your adaptation to Kazakhstani context? | 1,00 | 4,00 | 1,8069 | ,61573 | ,379 |
| What difficulties did you encounter in Kazakhstan (main problem)? | 1,00 | 4,00 | 1,8897 | ,97266 | ,946 |
| Do you find it difficult to find a balance between the culture of your origin and the culture of your country of residence? | 1,00 | 4,00 | 1,4069 | ,80361 | ,646 |
| Have you experienced pressure or faced negative attitudes because you are from another country? | 1,00 | 4,00 | 2,2897 | ,76301 | ,582 |
| Do you have any friends or support in Kazakhstan? | 1,00 | 3,00 | 2,1241 | ,57594 | ,332 |
| What aspects of the local culture have been the most challenging for you? | 1,00 | 4,00 | 2,4483 | 1,08609 | 1,180 |
| Has your time here affected your family relationships? | 1,00 | 4,00 | 2,4000 | 1,00277 | 1,006 |
| How do you deal with the emotional aspects of being physically separated from your family? | 1,00 | 5,00 | 2,6690 | 1,36451 | 1,862 |
| What challenges do you face in maintaining strong family relationships? | 1,00 | 4,00 | 1,9655 | 1,29322 | 1,672 |
| Does keeping in touch with your family help you better adapt? | 1,00 | 6,00 | 2,3958 | 1,90773 | 3,639 |
| How do you cope with the lack of physical intimacy with your family? | 1,00 | 4,00 | 1,9586 | ,90428 | ,818 |
| How often do you communicate with your family? | 1,00 | 4,00 | 2,1793 | 1,01152 | 1,023 |
| What means of communication do you prefer to use? | 1,00 | 4,00 | 1,5862 | ,82147 | ,675 |
| How do you deal with time zone differences when communicating with family or relatives living in another country? | 1,00 | 4,00 | 1,7586 | ,74792 | ,559 |
| What language situations do you find difficult? | 1,00 | 3,00 | 1,3931 | ,68004 | ,462 |
| Do you share your joys and sorrows with your loved ones? | 1,00 | 5,00 | 2,0276 | 1,29607 | 1,680 |
| Is it possible to openly discuss family problems in your family? | 1,00 | 5,00 | 1,9241 | 1,12474 | 1,265 |
| How did your family react to your decision to move to Kazakhstan for university studies? | 1,00 | 4,00 | 1,6759 | ,80694 | ,651 |
| How does your family support you in dealing with problems or difficulties? | 1,00 | 3,00 | 1,4966 | ,67828 | ,460 |
| Is your emotional well-being affected by the absence of your family? | 1,00 | 4,00 | 2,5310 | ,82542 | ,681 |
| What cultural traditions of your family do you actively support in Kazakhstan? | 1,00 | 4,00 | 1,8414 | ,75163 | ,565 |
| What cultural traditions of your native country do you actively support? | 1,00 | 4,00 | 2,2828 | 1,11619 | 1,246 |
| Do you like the food of the country you come from? | 1,00 | 4,00 | 2,2690 | 1,07532 | 1,156 |
| What types of support are useful for students from other countries in terms of maintaining family ties and adapting to a new culture? | 1,00 | 4,00 | 1,5310 | ,80842 | ,654 |
| How do you feel about your role in supporting and developing compatriots at the university where you are currently studying? | 1,00 | 4,00 | 1,8414 | ,81374 | ,662 |
| Do you plan to return to your country of origin? | 1,00 | 4,00 | 2,3103 | 1,07055 | 1,146 |
| What aspects of your new life do you value most? | 1,00 | 4,00 | 2,3931 | ,95231 | ,907 |
| How do you see the development of family relations in the future? | 1,00 | 4,00 | 1,5862 | ,80438 | ,647 |
| What year of study are you currently enrolled in? | 1,00 | 4,00 | 1,8621 | ,90998 | ,828 |
| What faculty are you currently enrolled in? | 1,00 | 6,00 | 1,9862 | 1,22467 | 1,500 |
| What is your mother tongue(s)? | 1,00 | 10,00 | 3,8414 | 2,38536 | 5,690 |
| What's your age? | 1,00 | 6,00 | 1,1517 | ,64905 | ,421 |
